# In-plane dipolar nano-antenna steers polariton waves at nanoscale

**DOI:** 10.1038/s41377-023-01284-2

**Published:** 2023-10-18

**Authors:** Huanjun Chen, Shaozhi Deng

**Affiliations:** State Key Laboratory of Optoelectronic Materials and Technologies, Guangdong Province Key Laboratory of Display Material and Technology, School of Electronics and Information Technology, Sun Yat-sen University, Guangzhou, 510275 China

**Keywords:** Nanophotonics and plasmonics, Metamaterials

## Abstract

Hyperbolic polaritons can be launched and guided into mirror-symmetric-broken trajectories using an in-plane dipolar nano-antenna, and this asymmetry can be configured by adjusting the polarization direction of the in-plane dipole moment.

Polaritons, the hybridized electromagnetic modes formed by coupling of incident light field with charged elementary excitations in matter, are able to confine the free-space light field down to the deep sub-wavelength regions, which can significantly enhance the light−matter interactions on nanoscale. Recently discovered van der Waals crystals (e.g., hexagonal boron nitride, α-MoO_3_, and α-V_2_O_5_)^1–4^ and low-symmetry polar crystals (e.g., CdWO_4_, β-Ga_2_O_3_)^5–7^ are demonstrated to sustain hyperbolic polaritons, where the polaritons propagating within these crystals experience an isofrequency surface of hyperboloid shape. The hyperbolic polaritons can exhibit ultrashort wavelengths, making them attractive for sub-wavelength light trapping and manipulation. In particular, the in-plane hyperbolic polaritons in some of these crystals (e.g., α-MoO_3_, α-V_2_O_5_, calcite, and β-Ga_2_O_3_), whose isofrequency surface in the two-dimensional (2D) plane has a hyperbolic geometry, can exhibit high polariton momentum (in principle toward infinity in some specific directions in the absence of loss) on the surface, thus opening up new avenues for manipulation and configuration of light flow at the 2D plane^8–11^. This can greatly facilitate design and fabrication of planar integrated photonic and optoelectronic devices and circuits.

Ideally, steering of in-plane polariton waves in a particular direction can achieve more focused and efficient electromagnetic wave light flow on the 2D plane. However, in current material systems, the dispersion of in-plane polariton waves typically exhibits mirror-symmetry, posing a significant challenge for achieving directional flow of the polaritons. This is because usually the excitation field contains momenta distributing isotropically inside the 2D plane. Each polariton wave will experience the same excitation efficiency, giving rise to a spreading circular wavefront (for in-plane isotropic crystals, left panel in Fig. 1) or beams with mirror-symmetry (for in-plane hyperbolic crystals, middle panel in Fig. 1). This challenge can be overcome by reducing the material’s symmetry and introducing a non-diagonal permittivity tensor. This modification disrupts the mirror symmetry of the isofrequency surfaces. However, one must address the significant propagation loss of polariton waves in low-symmetry crystals, which presents an additional obstacle. In a recent publication in *eLight*^12^, Peining Li and collaborators have successfully tackled this issue by employing a nano-antenna positioned on the surface of a high-symmetry crystal. The nano-antenna exhibits a robust in-plane dipolar oscillation, which serves as an excitation source for launching hyperbolic polariton waves (right panel in Fig. 1). These waves propagate without adhering to the mirror-symmetric propagation behavior typically observed in the basal plane of such a high-symmetry crystal.

**Fig. 1:**
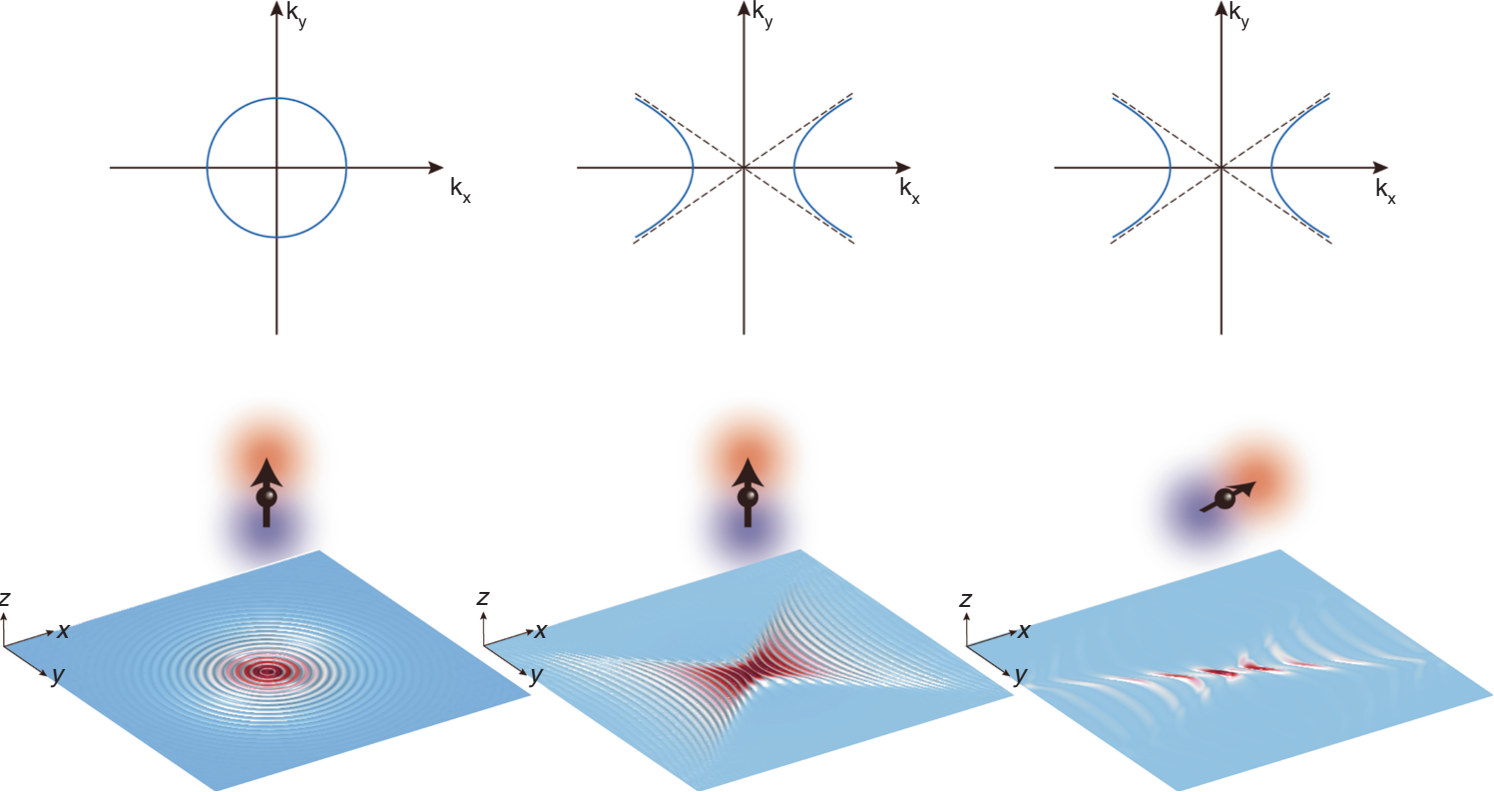
Launching of polaritons by a dipole source. Left panel: excitation of polaritons in a crystal with in-plane isotropic isofrequency contour (e.g., hexagonal boron nitride) by a *z*-orientated dipole source. Middle panel: excitation of polaritons in a crystal with in-plane hyperbolic isofrequency contour (e.g., α-MoO_3_, calcite) by a *z*-orientated dipole source. Right panel: excitation of polaritons in a crystal with in-plane hyperbolic isofrequency contour by an in-plane orientated dipole source

A prerequisite for the excitation of mirror-symmetric-breaking hyperbolic polaritons is the introduction of an in-plane asymmetric source field distribution. This requirement, as demonstrated in their study, can be achieved by integrating a gold disk nano-antenna onto the surface of a bulk calcite crystal. When excited by a vertically incident plane wave, the nano-antenna undergoes linearly polarized dipole oscillation in the horizontal direction and emits radiation with anisotropic characteristics within the crystal’s basal plane. In the momentum space, the radiation pattern exhibits maxima along the dipole axis and minima perpendicular to it. To effectively excite polariton waves in calcite, it is crucial for the momenta of the radiation from such a dipolar antenna to align with the in-plane wavevector distribution range of calcite polaritons, which is determined by the two asymptotes of the hyperbolic isofrequency contour (IFC). This overlap is necessary for the successful excitation of polariton waves in the desired direction. Without this alignment, the excitation of polariton waves in calcite would not occur. In this scenario, an effective method to excite mirror-symmetry-breaking polariton beams is by tilting the dipole axis of the nano-antenna relative to the asymptote of the in-plane IFC. This can be accomplished by rotating the polarization of the incident plane wave. When the minimum radiation direction of the dipole antenna aligns with one of the asymptotes, it leads to the observation of ray-like polariton propagation with a wavevector along the other asymptote. This phenomenon, which was defined as “symmetry-broken” state in Peining Li’s study, can be described as polariton steering, where energy flow is efficiently confined into one direction.

The team’s proposed approach allows for the precise steering of polariton beams in a specific direction on a high-symmetry crystal, overcoming the significant loss typically experienced within low-symmetry crystals hosting polaritons with intrinsic non-mirror-symmetry propagation behaviors. This advancement greatly enhances the high-efficiency transmission of energies and signals carried by these hybrid electromagnetic modes. One particularly exciting aspect of the team’s results is the ability to precisely control the propagation of hyperbolic polaritons under symmetry breaking. This control is achieved by carefully selecting the in-plane dipole orientation and operation frequency, which determines two asymptotes of the isofrequency contour (IFC). Consequently, the direction of polariton propagation can be intricately designed and controlled.

The simple yet robust method proposed by the team enables on-demand control of the polariton propagation at the nanoscale and over a broad spectral range. Looking forward, this will foster both of fundamental research and future possible applications. The unidirectional flow of polariton beams results in a significant confinement of electromagnetic fields and a notable acceleration of light–matter interactions in that direction. This approach offers opportunities for studying various nonlinear optics processes on a nanoscale. Additionally, it serves as an excellent test platform for imitating certain quantum optics experiments, such as the strong coupling between electromagnetic fields and quantum emitters, at room temperature and on a deep sub-wavelength level, which are beyond the reach of conventional cavity quantum electrodynamics experiments. In line with this study, an intriguing and attainable task is to deliberately design specific antenna structures to generate radiation sources with complex angular spectrum distributions. By exploiting the overlap between this angular spectrum distribution and the polariton IFC, it becomes possible to achieve more intricate polariton propagation trajectories.

This method is also expected to be applied in developing integrated photonics devices, such as nano-waveguides, optical switches, optical isolators, etc., thus promoting the development of optical computing, optical sensing, quantum optics, and other fields, especially in the mid-infrared and terahertz spectral range. In principle, the broken mirror symmetry of hyperbolic polaritons is essentially caused by the excitation of an in-plane electric dipole moment, rather than relying on the intrinsic hyperbolic dispersion properties of the materials. Therefore, the method can be extended to materials supporting other types of polaritons as well, such as plasmon polaritons in graphene, exciton polaritons in transition metal dichalcogenides, and magnon polaritons in magnetic crystals. This allows for the design and fabrication of hybrid materials and related photonic and optoelectronic devices with multiple functionalities derived from different materials. Overall, the present work simplifies both the principles and architecture for the manipulation of polariton propagation, and opens up various exciting opportunities for further exploration.
